# The Numerical Analysis of Force and Comparison of Pulse Magnet and Electromagnetic Forming Coil

**DOI:** 10.3390/ma16175828

**Published:** 2023-08-25

**Authors:** Yanxin Li, Bo Tang, Yiliang Lv, Qi Xiong, Xiang Zhao

**Affiliations:** 1 College of Electrical Engineering & New Energy, China Three Gorges University, Yichang 443002, China; lyxamaz800621@163.com (Y.L.); tangboemail@sina.com (B.T.); zhaoxiang199811@163.com (X.Z.); 2 Hubei Provincial Engineering Technology Research Center for Power Transmission Line, China Three Gorges University, Yichang 443002, China; 3 Wuhan National High Magnetic Field Center, Huazhong University of Science and Technology, Wuhan 430074, China; yilianglv@hust.edu.cn

**Keywords:** pulsed magnet, forming coil, von Mises stress, reinforcing material

## Abstract

As an important energy conversion component in electromagnetic-forming technology, the coil is subjected to great internal stress and is easy to break. The geometric structure and winding process of the forming coil draw on the research results of pulsed magnets. However, the two use conditions are different. It is very important to clarify the force difference between the two for the design of the forming coil. In this paper, the numerical model of an aluminum alloy (AA1060-O) is established, and the difference in force between the pulse magnet and forming coil with the same size in time and space under different working conditions is analyzed. A two-dimensional fully coupled finite element model consisting of circuit, magnetic field, and solid mechanics is established and used to determine the key parts of the coil force. It is found that the von Mises stress of the forming coil is greater than that of the pulsed magnet under the same circuit parameters and geometric structure. In the electromagnetic forming of the tube, the glass fiber is subjected to a large stress. In addition, the stress of glass fiber under the condition of tube necking is about 2 times that of pulsed magnet. When the voltage is increased, the failure of the middle part of the glass fiber causes the coil to break. In the electromagnetic forming of the sheet, the coil skeleton is subjected to large stress, and its upper end failure causes the coil to break. Therefore, new design ideas for forming coils under different working conditions are proposed.

## 1. Introduction

Electromagnetic forming (EMF) is a high-speed forming technology widely used in light alloy tubes and sheets in recent years [1]. Compared with the traditional process, electromagnetic forming has the characteristics of a high strain rate and no pollution [2]. As an important energy conversion component in the system, the coil works in the extreme electromagnetic environment of the pulsed strong magnetic field [3]. The high-strength magnetic field generated by the coil interacts with the current in the conductor to generate a huge electromagnetic force. The coil works in a magnetic field of more than 20 T for a long time, and the material gradually deforms to cause a rupture [4]. The strength of the coil is affected by its structure, material, and manufacturing process. The early electromagnetic forming coil is mainly a flat plate type, using copper as a conductor, which is directly used after cutting by a wire cutting machine. The mechanical strength of the conductor material is difficult to resist the huge magnetic stress and eventually ruptures [5].

In 2011, the Pulsed High Magnetic Field Science Center of Huazhong University of Science and Technology introduced the winding technology of pulsed magnets into the electromagnetic forming coil [6]. Solenoid coils (referred to as the forming coil) began to be widely used. The interlayer reinforcement material of the coil conductor generally uses Zylon, and the outer layer reinforcement uses glass fiber [7]. Finally, the whole coil is reinforced with epoxy resin and fixed [8]. The forming coil adopts the reinforcement technology of the pulse magnet to improve the overall strength. However, unlike pulsed magnets, forming coils are placed in different positions inside and outside the tube when used for tube bulging and necking [9,10]. It is placed at the end of the workpiece during electromagnetic flanging [11]. It is placed on the upper and lower sides of the workpiece during sheet forming [12]. In the electromagnetic-assisted forming and incremental forming, the coil is placed in the specified position [13,14]. Soni et al. designed the deformation experiment of the AA6061 aluminum tube, and it was found that the circuit parameters and the deformation of the workpiece would affect the performance of the coil [15]. Elsayed et al. discussed the types of coils and the influences of various parameters on the coils and introduced methods to improve the quality of the coils [16]. Lim et al. studied the von Mises stress and effective plastic strain of the workpiece during the forming process [17]. Doley et al. established the free forming of an AA6061 aluminum alloy sheet with a thickness of 1mm by using finite element software. It is found that the failure area of the workpiece in the simulation results was consistent with the experiment [18].

Under these different working conditions, the force of the forming coil is not the same as that of the pulsed magnet, and the structural strength theory related to the pulsed magnet cannot be copied. At the same time, it was also found in the experiment that the coil would fail and break. Therefore, only by clarifying the stress difference between the two can the forming coil be designed in a targeted manner and its service life be prolonged. Based on this, the numerical model of tube and sheet forming of an aluminum alloy (AA1060-O) is established in this paper. The force difference between the pulsed magnet and the forming coil in time and space is analyzed in detail, and the key force parts of the coil are distinguished. A new design idea is proposed for the forming coil under different working conditions.

## 2. Methods

**Figure 1:**
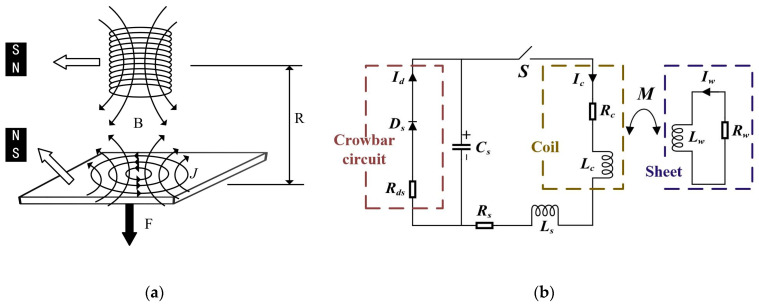
Schematic diagram of electromagnetic-forming principle. (**a**) Lenz’s law; (**b**) equivalent circuit diagram.

Electromagnetic-forming technology is to generate an induced magnetic field by introducing a pulsed high current into the coil and then generating an induced eddy current in the workpiece, forming a strong electromagnetic force to cause plastic deformation of the workpiece [19], as shown in Figure 1.

The discharge circuit of the electromagnetic-forming system is mostly composed of RLC components and forming coils in series. A crowbar circuit is connected in parallel in the circuit to ensure that the discharge current does not oscillate [20].

Electromagnetic forming is the interaction between the magnetic field and eddy current in the workpiece, which generates electromagnetic force to drive the deformation of the workpiece. The electromagnetic force is obtained by the following formula.

(1) $$F=J_{e}\times B$$

In the formula, $J_{e}$ and B are the induced eddy current density and magnetic flux density on the workpiece. Due to the influence of the structure of the solenoid coil, the eddy current density $J_{e}$ is only the circumferential component $J_{ephi}$. They can be expressed as follows:

(2) $$\left\{\begin{array}{l} F_{r}=J_{ephi}\times B_{z}\\ F_{z}=-J_{ephi}\times B_{r} \end{array}\right.$$

In the formula, $B_{r}$ and $B_{z}$ are the radial and axial components of the magnetic flux density, respectively. $F_{r}$ and $F_{z}$ are the radial and axial components of the electromagnetic force, respectively. Negative indicates repulsive force.

This paper mainly discusses the force of the coil under the single pulse unidirectional repulsive electromagnetic force.

The elastoplastic mechanical model of pulsed magnets is a static elastoplastic mechanical analysis based on the small deformation assumption. During the deformation process of the pulsed magnet, the maximum displacement is much smaller than its geometric width. There is almost no acceleration during the deformation process. In the process of electromagnetic forming, although the deformation of the workpiece is very severe, the forming coil is generally fixed by the tooling, and there is no accelerated motion. Therefore, the model is suitable for the internal force analysis of the two. The equilibrium equation is:

(3) f+∇⋅σ=0

In the formula, f is the electromagnetic force, and there is f=0 in the reinforcement layer. σ is the stress tensor.

When the material ruptures is the focus of the coil force analysis. Based on many studies, the stress failure criteria of orthotropic elastic materials, such as reinforcement materials, are determined. The maximum von Mises equivalent stress of the material is judged by whether it exceeds its ultimate tensile strength [21]. At room temperature, the ultimate strength of glass fiber is 3000 MPa, and the ultimate strength of epoxy resin is 320 MPa [22].

According to the von Mises yield criterion.

(4) $$\sigma_{M}=\sqrt{\frac{1}{2}\left({\left(\sigma_{r}-\sigma_{t}\right)}^{2}+{\left(\sigma_{r}-\sigma_{z}\right)}^{2}+{\left(\sigma_{t}-\sigma_{z}\right)}^{2}\right)}$$

In the formula, $\sigma_{0}={\left(\sigma_{r}-\sigma_{t}\right)}^{2}+{\left(\sigma_{r}-\sigma_{z}\right)}^{2}+{\left(\sigma_{t}-\sigma_{z}\right)}^{2}$ is the initial yield stress of the material. $\sigma_{M}$ is the equivalent stress of plastic deformation. $\sigma_{r},\;\sigma_{t},\;\sigma_{z}$ are the stresses along the radial, circumferential, and axial directions, respectively.

(5) $$\varepsilon_{M}=\frac{1}{1+v}\sqrt{\frac{1}{2}\left({\left(\varepsilon_{r}-\varepsilon_{t}\right)}^{2}+{\left(\varepsilon_{r}-\varepsilon_{z}\right)}^{2}+{\left(\varepsilon_{t}-\varepsilon_{z}\right)}^{2}\right)}$$

In the formula, $\varepsilon_{M}$ is the equivalent strain of plastic deformation. v is the Poisson’s ratio of the material entering the plastic stage. $\varepsilon_{r},\;\varepsilon_{t},\;\varepsilon_{z}$ are radial, circumferential, and axial strains, respectively.

After the material enters the plastic deformation stage, the equivalent stress and strain are very complex. By setting the boundary conditions of the coil, the equivalent stress and strain can be calculated more accurately. Take single-layer conductors and reinforcement materials as an example. The radial stress and circumferential strain on the outer surface of the inner sublayer should be equal to the radial stress and circumferential strain on the inner surface of the outer sublayer. They can be expressed as follows:

(6) $$\left\{\begin{array}{l} \sigma_{ri}\left(i+1\right)=\sigma_{r0}\left(i\right)\\ \varepsilon_{ti}\left(i+1\right)=\varepsilon_{r0}\left(i\right) \end{array}\right.$$

In the formula, $\sigma_{ri}\left(i\right),\sigma_{r0}\left(i\right),\varepsilon_{ti}\left(i\right),\varepsilon_{to}\left(i\right)$ are the radial stress and hoop strain on the inner and outer surfaces of the i-sublayer, respectively.

The outermost sublayer of the coil is a free surface, and the radial stress should be zero. The radial magnetic field component produces an axial electromagnetic force in the conductor. The sum of the product of the axial stress and the area of each sublayer should be equal to the electromagnetic force, expressed as:

(7) $$\left\{\begin{array}{l} \sigma_{r}\left(2M\right)=0\\ \sum_{i=1}^{2M}\sigma_{z}\left(i\right)S\left(i\right)=F_{z} \end{array}\right.$$

At the same time, the press imposes a fixed constraint on the upper surface of the coil.

## 3. Numerical Simulation and Materials

To explore the stress of pulsed magnets and forming coils of the same size under different working conditions. In this paper, the finite element model with experimental results in Refs. [23,24] is used. The stress state of the coil in the electromagnetic forming of tubes and sheets is analyzed.

**Figure 2:**
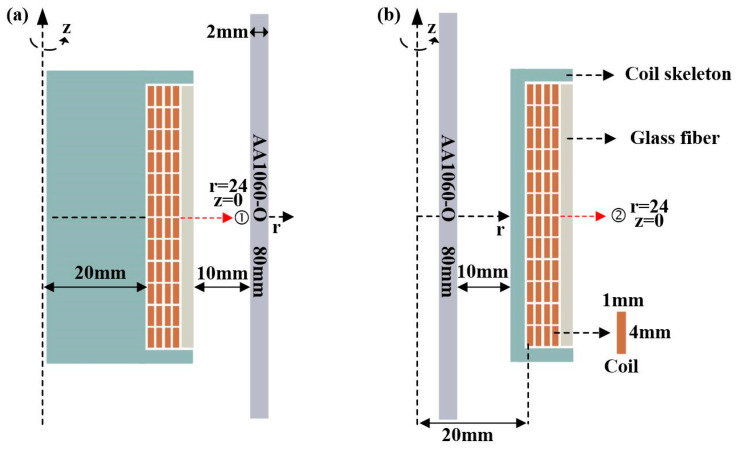
Geometrized structure graph of tube electromagnetic-forming scheme. (**a**) Tube bulging; (**b**) tube necking.

In the electromagnetic-forming scheme of the tube, a single turn coil of 1 mm × 4 mm is used. The reinforcement material is a S2 glass fiber. S-type has high strength, good corrosion resistance, and heat resistance [25]. During the electromagnetic bulging and necking of the tube, the distance between the tube and the conductor is always kept at 12 mm. The detailed geometric dimensions are shown in Figure 2.

**Figure 3:**
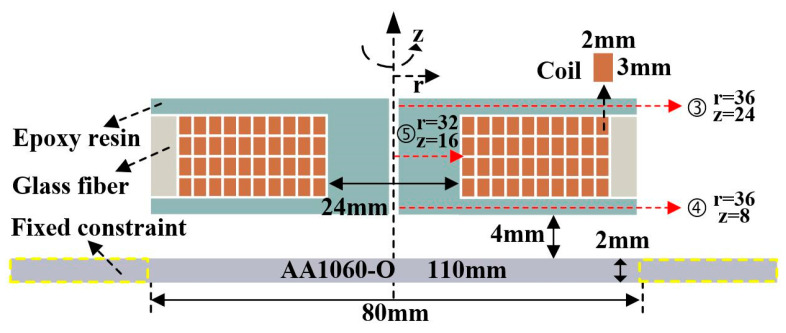
Geometric structure of sheet electromagnetic-forming scheme.

Based on the geometric design of glass fiber, the middle position of glass fiber is selected for subsequent analysis. In the electromagnetic-forming scheme of the sheet, a single turn coil of 2 mm × 3 mm is used. Using S2 glass fiber reinforcement, a 3240-epoxy board was selected for packaging. The geometric dimensions are shown in Figure 3.

The detailed parameters of the geometric structure, circuit and material of the electromagnetic-forming scheme are shown in Table 1.

**Figure 4:**
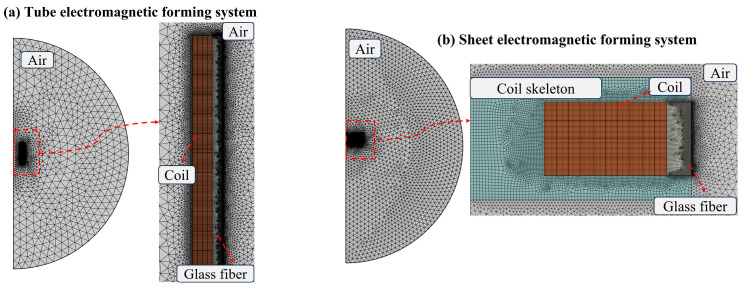
Modeling and meshing of electromagnetic-forming scheme. (**a**) The meshing of tube forming; (**b**) The meshing of sheet forming.

To reduce the calculation amount of the simulation, the calculation error is not affected. The model is divided by the grid of conventional unit size. In the tube forming-scheme, the maximum element of the grid is 26.8 mm, the minimum element is 0.12 mm, and the average element mass is 0.8913. In the sheet-forming scheme, the maximum element of the grid is 14.2 mm, the minimum element is 0.18 mm, and the average element mass is 0.9164. Figure 4 is the grid division.

## 4. Results Analysis and Discussion

**Figure 5:**
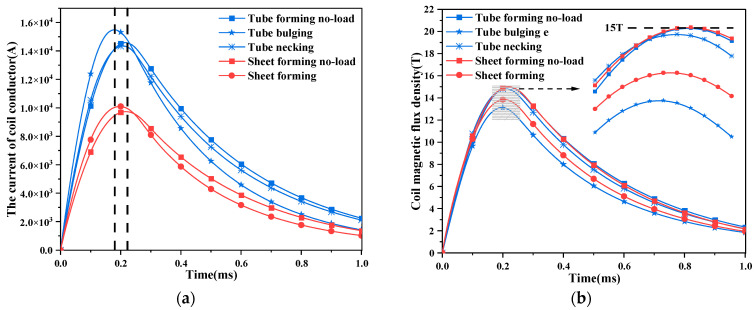
The time state quantity of coil no-load and workpiece forming. (**a**) The state quantity of coil current; (**b**) state quantity of coil magnetic flux density.

The coil geometry and position of the two electromagnetic-forming schemes of tube and sheet are different. The forces of the two cases must be inconsistent. Therefore, the force of the coil is analyzed according to different forming schemes. Due to the use of pulsed magnets as field sources, the no-load test of coils in electromagnetic forming is equivalent to pulsed magnets. Based on the specification and premise of the above design scheme, the current state and magnetic flux density state of the pulsed magnet and the forming coil are obtained, as shown in Figure 5.

The coil current reaches a peak between 0.18 ms–0.22 ms. Currently, the forming requirements are completed. The coil current increases after adding the workpiece. This shows that the induced magnetic field of the workpiece also induces eddy currents in the coil conductor, which increases the coil current.

After many parameter debugs, the maximum magnetic flux density generated by the pulsed magnet in both cases is 15 T. It is found that the maximum magnetic flux density is less than 15 T after adding the workpiece. This is because the deformation of the workpiece has a reaction force on the coil, which changes the coupling relationship of the magnetic field and weakens the magnetic field.

Aiming at the force difference between the pulsed magnet and the forming coil, this paper analyzes it in detail from time and space.

### 4.1. The Force Analysis of Tube Forming Coil under No-Load and Load

#### 4.1.1. Comparison of Force on Time Distribution

**Figure 6:**
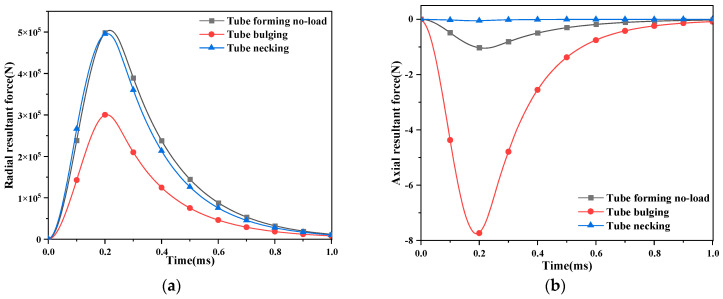
The change in the force of the coil conductor with time. (**a**) The radial resultant force; (**b**) the axial resultant force.

Figure 6 shows the change in the axial and radial resultant force of the coil conductor with time to judge the main stress position of the coil. If it is mainly subjected to radial force, it is transferred to the glass fiber. If it is mainly subjected to axial force, it is transmitted to the coil skeleton.

**Figure 7:**
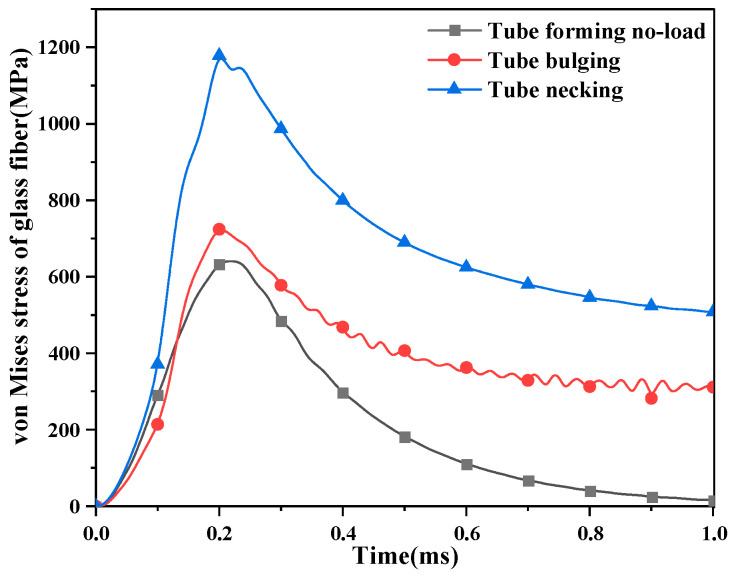
The change in the von Mises stress of glass fiber with time.

In the electromagnetic-forming scheme of the tube, the axial force change in the pulse magnet and the forming coil is not more than 8 N, which can be ignored. The radial resultant force of the pulsed magnet is 5 × 10^5^ N, which decreases slightly after adding the workpiece. The rupture of the coil is caused by the excessive radial force, and the stress of the glass fiber should be discussed. Figure 7 shows the change in the von Mises stress of glass fiber with time.

The change in von Mises stress on glass fiber with time conforms to the trend in coil current changing with time. The stress reaches the maximum value at the peak time of the coil current.

#### 4.1.2. Comparison of Force on the Spatial Distribution

**Figure 8:**
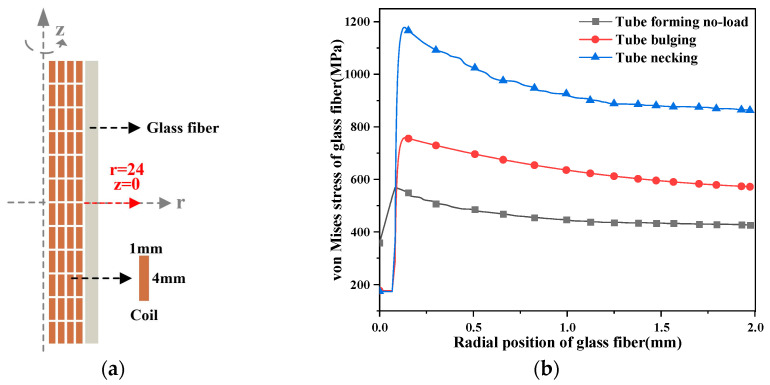
The change in the force in the middle of the glass fiber in the radial position. (**a**) The geometric coordinates of the middle of the glass fiber; (**b**) variation in the von Mises stress with time.

As mentioned in the previous section, the von Mises stress in the middle position of the glass fiber changes with the radial position, as shown in Figure 8.

The von Mises stress in the middle of the glass fiber increases rapidly to the maximum within 0.2 mm, and the stress gradually decreases as the radial position moves outward. Although the changing trend is the same, the values are quite different. The von Mises stress of the pulsed magnet reaches the maximum value of 589 MPa at 0.14 mm, the maximum value of 770 MPa at 0.09 mm during tube bulging, and the maximum value of 1210 MPa at 0.12 mm during tube shrinkage. The forming coil is subjected to greater stress.

**Figure 9:**
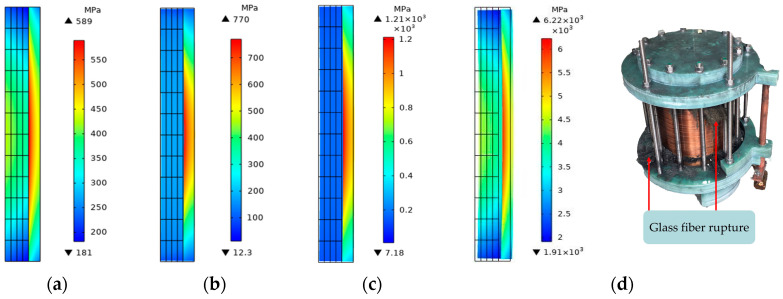
Two-dimensional plane distribution of von Mises stress on glass fiber. (**a**) Pulsed magnet; (**b**) tube bulging; (**c**) tube necking; (**d**) glass fiber failure causes coil rupture.

As shown in Figure 9, the von Mises stress on the coil will not cause the failure of the glass fiber. Therefore, without changing other circuit parameters, only the power supply voltage is increased to 26 kV, and the force of the failure glass fiber is obtained.

The von Mises stress on the glass fiber reached 6220 MPa when the coil ruptured, exceeding its ultimate strength of 3000 MPa. Therefore, when designing the coil, it is recommended to wind the high-strength glass fiber near the conductor side and then wind the ordinary glass fiber until the outermost side. Alternate windings of glass fibers with different strengths can ensure the highest strengths at the maximum force of the coil and reduce the cost.

### 4.2. The Force Analysis of Sheet Forming Coil under No-Load and Load Conditions

#### 4.2.1. Comparison of Force on Time Distribution

**Figure 10:**
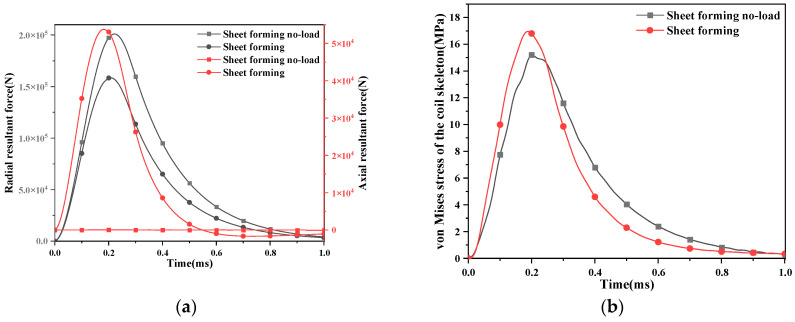
The force of the conductor and the coil skeleton. (**a**) The change in the axial and radial resultant force of the conductor with time; (**b**) variation in von Mises stress on the coil skeleton with time.

In the electromagnetic-forming scheme of the sheet, the radial force of the pulsed magnet is greater than that of the forming coil, which is 2.01 × 10^5^ N. The front section shows that the radial force at this time will not deform the glass fiber. The axial force of the pulsed magnet is 73 N, which increases to 5.4 × 10^4^ N after the workpiece is added, and the axial force increases sharply. Therefore, the stress of the coil skeleton should be discussed. As shown in Figure 10, the change in von Mises stress on the coil skeleton with time conforms to the trend in coil current changing with time. 

#### 4.2.2. Comparison of Force on the Spatial Distribution

**Figure 11:**
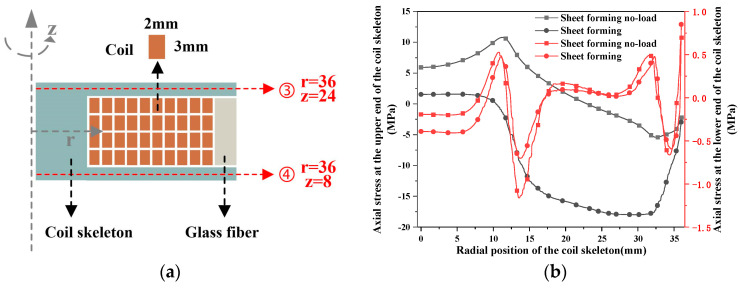
The variation in the stress of the coil skeleton with the radial position. (**a**) The geometric coordinates of the upper and lower ends of the coil skeleton; (**b**) the change in axial stress on the coil skeleton in the radial position of the upper and lower ends.

Based on the analysis of the previous section, the upper, lower and middle parts of the coil skeleton are taken. As shown in Figure 11, the change of axial stress with radial position is analyzed.

**Figure 12:**
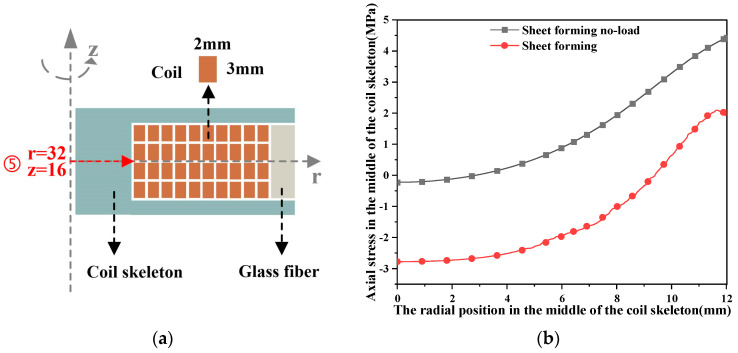
The variation in the stress of the coil skeleton with the radial position. (**a**) The geometric position of the middle of the coil skeleton; (**b**) the variation in axial stress of the coil skeleton in the middle radial position.

The axial stress change in the lower end of the coil skeleton of the pulsed magnet and the forming coil is not more than 1 MPa, so it is not discussed. The axial stress on the upper end of the coil skeleton of the formed coil is about 3 times that of the pulsed magnet. Figure 12 shows the axial stress in the middle of the coil skeleton, which does not change more than 3 MPa and is not discussed here. In the electromagnetic forming of the sheet, the coil rupture is caused by the large axial stress on the upper end of the coil skeleton.

**Figure 13:**
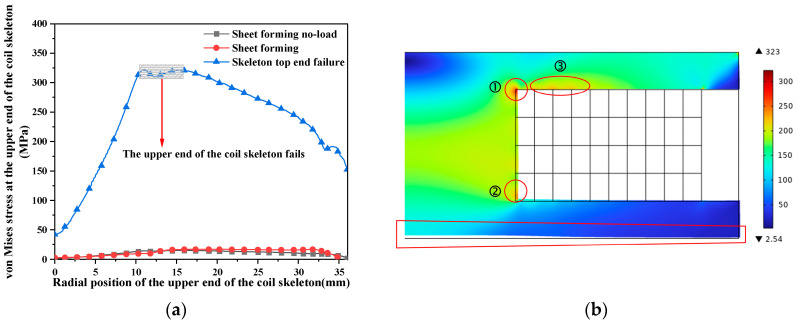
Contrast diagram of the stress of the coil skeleton. (**a**) von Mises stress on the failed and normal coil skeletons; (**b**) von Mises stress distribution of the failed coil skeleton.

As shown in Figure 13, the von Mises stress on the upper end of the coil skeleton increases first and then decreases in the radial position. The maximum von Mises stress at the upper end of the coil skeleton of the pulsed magnet is 14.8 MPa at 15.5 mm, and the maximum von Mises stress at the upper end of the coil skeleton of the formed coil is 17.1 MPa at 12.1 mm. At this time, the coil skeleton did not fail. Therefore, without changing other circuit parameters, only the power supply voltage is increased to 12 kV, and the force of the deformed the coil skeleton is obtained.

When the coil fails or even deforms, the von Mises stress of the coil skeleton is 323 MPa, exceeding its ultimate tensile strength of 320 MPa. As the upper end of the coil skeleton is fixed by the press during the experiment, the boundary conditions of the fixed constraint are set at the upper end of the coil skeleton during modeling. Therefore, the lower end of the coil skeleton in the diagram has been deformed upwards, but the upper end does not exceed the boundary. In fact, the upper end of the coil skeleton has been broken. In the diagram, there are evidently three areas with large stress, and the von Mises stress of area ① at the upper end of the coil skeleton is the largest. At the same time, the force area at the upper end of the coil skeleton is larger, which begins to fail and leads to the overall rupture of the coil. Therefore, when designing the coil, it is recommended to thicken the upper end of the coil skeleton or wind a layer of glass fiber around the upper end of the conductor to reduce the stress transmitted to the upper end of the coil skeleton.

## 5. Conclusions and Prospects

In this paper, the force process of the electromagnetic forming coil and pulse magnet was analyzed first. Then, the force of the coil and its reinforcement material under different forming conditions was analyzed in detail from time and space through experiments and simulations. Finally, some suggestions were put forward for coil reinforcement designs under different working conditions. The main conclusions were as follows:

1. Under the same circuit parameters, the von Mises stress of the formed coil was greater than that of the pulsed magnet. The force of the tube during the necking was greater than that of the tube bulging, which was about 2 times that of the pulsed magnet;
2. Under different working conditions, the force direction and position of the forming coil were different. In the electromagnetic forming of the tube, the coil was subjected to a large radial force, and the failure of the middle part of the glass fiber caused the rupture. In the electromagnetic forming of the sheet, the coil was subjected to a large axial force, and the upper end of the coil skeleton was broken due to failure;
3. Coil design recommendations. In the electromagnetic forming of the tube, the glass fiber with higher strength was wound near the coil conductor, and the middle part of the glass fiber was thickened. In the electromagnetic forming of the sheet, the glass fiber was wound at the connection between the upper end of the coil skeleton and the conductor, and the upper end of the skeleton was thickened.

In practice, the production costs of pulsed magnets and forming coils are high, and a coil is used multiple times. The flow of the coil material is superimposed on the microscopic flow, and the plastic deformation cannot be restored. The coil is easy to break after long-term use. Therefore, regular quality inspection of the coil is very important. If it can be replaced or reinforced again in time, the service life of the coil will be prolonged, and the production cost will be saved.

## Figures and Tables

**Table 1:** Geometric parameters, circuit parameters, and material parameters.

Components	Parameters	Numerical Value of Forming Scheme	
		Tube Forming	Sheet Forming
Pulsed magnet	Maximum magnetic flux density	15 T	15 T
Power supply and line	Capacitor parameters	320 µF	320 µF
	Discharge voltage	8 kV/26 kV	6 kV/12 kV
	Line resistance	75 mΩ	130 mΩ
	Line inductance	9 µH	20 µH
	Continuous current resistance	65 mΩ	55 mΩ
Coil (Copper)	Coil inner radius	12 mm	20 mm
	Coil outer radius	32 mm	24 mm
	Coil height	12 mm	48 mm
	Number of turns of coil	10 n	4 n
	Conductivity	5.998 × 10^7^ [S/m]	
	Mass density	8960 [kg/m^3^]	
	Strain rate strength coefficient	0.025	
	Young’s modulus	110 GPa	
	Poisson’s ratio	0.35	
Workpiece (AA1060-O)	Workpiece radius	36 mm/6 mm	40 mm
	Workpiece thickness	2 mm	2 mm
	Distance between coil and workpiece	12 mm	4 mm
	Conductivity	3.72 × 10^7^ [S/m]	
	Mass density	2710 [kg/m^3^]	
	Young’s modulus	69 GPa	
	Poisson’s ratio	0.33	
Epoxy resin (3240)	Mass density	1800 [kg/m^3^]	
	Young’s modulus	{2, 5, 2} GPa	
	Poisson’s ratio	0.35	
Glass fiber (S2)	Mass density	2550 [kg/m^3^]	
	Young’s modulus	{3, 230, 3} GPa	
	Poisson’s ratio	0.3	

## Data Availability

The data reported in this research are available from the corresponding author upon request.
